# Family doctors’ attitudes toward peer support programs for type 2 diabetes and/or coronary artery disease: an exploratory survey among German practitioners

**DOI:** 10.1186/s12875-022-01827-3

**Published:** 2022-08-31

**Authors:** K. Majjouti, L. Küppers, A. Thielmann, M. Redaélli, F. Vitinius, C. Funke, I. van der Arend, L. Pilic, M. Hessbrügge, S. Stock, B. Weltermann, D. Wild

**Affiliations:** 1 Institute of General Practice and Family Medicine, University Hospital Bonn, Bonn University, Bonn, Germany; 2 Institute for Health Economics and Clinical Epidemiology, University Hospital of Cologne, Cologne, Germany; 3 Department of Psychosomatics and Psychotherapy, Faculty of Medicine, University Hospital Cologne, University of Cologne, Cologne, Germany; 4 Institute of General Practice, Heinrich-Heine-University of Düsseldorf, Duesseldorf, Germany; 5 Teaching Area General Medicine RWTH Aachen University, Aachen, Germany; 6 Institute of General Practice, Faculty of Medicine, University Hospital Cologne, Cologne, Germany; 7 Institute for General Medicine, University Hospital Essen, University of Duisburg-Essen, Essen, Germany

**Keywords:** Peer support program, Type 2 diabetes, Coronary artery disease, Primary care, Health behaviour change, Peer-education, Patient empowerment, Self-management

## Abstract

**Background:**

Type 2 diabetes (T2D) and coronary artery disease (CAD) are chronic illnesses where adherence to a healthy lifestyle is crucial. If organisational and cultural factors are well managed, Peer support programs (PSP) can improve self-management, quality of life, and health outcomes. In preparation for launching a PSP, we surveyed family doctors (FD) about their attitudes toward such a program and about potential barriers, and facilitators.

**Methods:**

In March 2020 we surveyed 896 FDs from five university teaching practice networks in North-Rhine Westphalia, Germany, via an anonymous web-based survey. The questionnaire addressed details of PSPs, including suitable patients and FDs‘role. Data were analysed using descriptive and inferential statistics; qualitative material underwent content analysis by two researchers.

**Results:**

A total of 165 FDs responded (response rate: 18.4%), 97% were practice owners. Respondents viewed PSPs positively (T2D: 92.0%, CAD 89.9%), especially for patients with poor self-structuring (82.7%), low motivation (76.3%) and few social contacts (67.6%). On average, FDs were able to identify 4.0 ± 3.2 patients as potential group leaders. Major facilitators reported included motivation by peers (92.5%), exercise (79.1%), and social contacts (70.1%). Waning interest over time (73.1%) and poor motivation (70.9%) were considered barriers. The majority of FDs would recommend PSPs to their patients (89.5%). They considered such a program a valuable addition to current care (79.7%). The percentage of FDs’ who expected long-term benefits for their workload was relatively low (37.6%).

**Conclusions:**

In an exploratory survey among German FDs on PSPs, respondents viewed PSPs as a valuable add-on for T2D and CAD patients, while not expecting a positive impact on their workload. Communication with FDs on PSPs may need to highlight anticipated implementation outcomes such as benefits of PSPs to the practice.

**Supplementary Information:**

The online version contains supplementary material available at 10.1186/s12875-022-01827-3.

## Introduction

Peer support programs (PSP) are an emerging approach that combines peer support with a structured program of education and assistance to improve emotional and social support, daily health management, and links to clinical care [1]. Programs based on peer support are an innovative method to improve patients’ quality of life and well-being as well as clinically important outcomes. Two recent systematic reviews of self-management interventions for diabetic patients found significant effects on glycated hemoglobin levels [2, 3] as well as self-efficacy and the frequency of emergency room visits [3], Peer support interventions have also been shown to reduce blood pressure ([4]; reduction of systolic blood pressure by 2.07 mmHg (CI 0.35–3.79 mmHg). Further results underlining the effectiveness of peer support were reported by Wing et al. [5] who demonstrated significant loss of weight (− 4 kg, CI − 5 to − 3, *p* < 0.001) and a decrease in waist circumference (− 3.2 cm, CI − 3.9 to − 2.4, *p* < 0.001). These exciting findings emphasise the promising role that peer support could play in future healthcare.

Peer support is defined as groups of individuals with similar conditions (and thus similar experiential knowledge) who mutually support each other to achieve long-term recovery from their health-related problems [5]. In contrast to traditional self-help groups, peer support programs are more structured and include skills training and lifestyle support as well as follow-up [6]. Varying models of such programs exist worldwide. They range from phone- or web-based approaches to professional-led and/or peer-led group meeting programs [6, 7]. PSPs complement traditional health care services by providing frequent, continuous, accessible, and flexible support to ensure adherence to lifestyle changes. They foster understanding and trust in health care providers and services, even among groups with poor access to health care [8, 9].

Most studies on PSPs have been conducted in the developing world or in English-speaking countries. It is unclear whether they can also be implemented successfully in high-income European countries. Given worldwide shortages in health care professionals [10] and problems of rising health care costs, a structured “lay person-integrating support system” may be a cost-effective, complementary structure within modern health care systems [11]. A recent review of peer education and counselling found that such interventions can be effective if organisational and cultural aspects are well managed [12] . Provider referral to PSPs is a crucial step in patients’ participation; both provider referral as well as organisational climate and support towards new programmes are important determinants at the “meso”-level of implementation [13].

.A publicly-funded PSP (P-SUP) is currently being developed for the North Rhine region, one of the most densely populated regions in Germany. This program is embedded in Germany‘s existing Disease Management Programs (DMPs) for patients with CAD and T2D. DMPs are structured treatment programs that aim to improve medical treatment, patients disease management skills, and quality of life. Besides quarterly consultations and medical examinations, the program offers special training courses for patients [14]. However,, DMPs alone lack sufficient self-management training [15]. As a new intervention in healthcare, PSPs depend on FDs referring patients [16]. Therefore, and in preparation for the new PSP, we conducted an anonymous web-based survey among FDs to assess their attitudes toward PSPs and their opinions regarding potential barriers to and facilitators for program implementation.

## Methods

### Study design and population

We performed an anonymous web-based survey among all primary care teaching practices (*n* = 896) of the Universities of Bonn, Duesseldorf, Cologne, Essen and Aachen.

#### Subject of questionnaire: peer support program and peer support group leaders

The questionnaire provided a brief introduction to the P-SUP intervention. Characteristics of the program are:

- Joint physical activity and support in peer groups,
- Online content and regional networking opportunities,
- Personalised feedback reports (along with regular DMP reports),
- Telephone coaching (needs-based),
- Selection of group leaders and recommendation to participate by FDs.

Peer Support Group Leaders (PSG-L) are patients who receive tailored training. This training integrates information on T2D and CAD, a healthy life style (diet, exercise), and group communication. PSG-L structure group meetings using the training materials provided, act as a role model and serve as a contact to the research team.

### Survey instrument

The online survey was constructed and administered using the professional online survey tool SoSci Survey. The full text of the survey is available in the Appendix (Supplementary Table S6). It consisted of 46 questions across eight categories. The content was developed on the basis of a literature review using the electronic research databases PubMed and Medline. The review was completed from March to April 2020. Based on our literature review, we included the following domains: peer support [17, 18], peer leaders ( [19–21], peer support training [20], health behaviour change [17, 22] and self-management of chronic diseases [22]. The first part of the questionnaire elicited sociodemographic and professional characteristics. The second part detailed various elements of the planned PSP including aspects related to the role of FDs. The survey applied various answer formats including Likert scales and rankings. In order to assess the attitude toward this largely novel concept, we offered free-text options and multiple choice questions. The following items were analysed:

- FDs‘experience with and communication regarding self-help groups (SHGs):
  - Patients‘requests for information on SHGs,
  - FD knowledge of SHGs in the area,
  - Usual practice of referring patients to SHGs.
- Demand for peer support programs from patients with CAD and/or T2D:
  - Degree to which available lay support meets patients‘needs,
  - Perceived benefit of peer support groups (PSGs),
  - Factors for recommending PSPs.
- Opinions regarding the optimal composition of PSGs:
  - Social background, medical aspects, organisational details.
- Target group for participation in a PSP:
  - Recommendation to all or only to selected patients,
  - Patient groups/types that would benefit most from PSPs.
- Identifying potential PSG-Ls:
  - Characteristics of potential PSG-Ls,
  - Number of potential PSG-Ls in the practice.
- Expected benefits of PSPs:
  - Potential long-term benefits for the practice,
  - Added-value of integrating PSPs into regular care,
  - Usefulness of PSPs,
  - Likelihood of recommending PSPs to own patients.
- Barriers and facilitators:
  - Aspects of the program considered beneficial, e.g., initiating social contacts or joint exercise activities,
  - Main barriers to implementation, e.g., lack of group cohesion or high drop-out rate,
  - FDs‘willingness to participate in PSPs as experts or seminar hosts.
- What additional information on PSPs respondents would like.

The questionnaire was developed and pre-tested in the multi-disciplinary research team with academic physicians from the fields of family medicine and psychosomatic medicine as well as scientists from public health, economy, sports and rehabilitation. All authors participated in the pre-test and refinement process of the instrument. As a results of the pretest, two items were rephrased to make them more comprehensible and then re-tested until all ambiguities were resolved.

### Data collection

FDs were invited by email and asked to complete the questionnaire within 2 weeks. The survey period started in early March 2020 at the beginning of the COVID-19 pandemic in Germany. Two reminders were sent after intervals of 2 and 5 weeks.

### Analysis of quantitative and qualitative data

The evaluation of the data was based on descriptive and inferential statistics. For the descriptive analysis, frequencies and percentages were used for qualitative variables, whereas quantitative variables were expressed as means and standard deviation (±SD). Regarding inferential statistics, a multivariable logistic regression model was employed after testing the model fitness using the Hosmer-Lemeshow goodness of fit test in order to examine the relationship between FD’s openness to PSPs with respect to age, practice setting, and experience with SHGs. Covariates were chosen based on the authors’ experience. The strength of association was determined by calculating crude odds ratio (OR) along with 95% confidence intervals (CI). The Regression analysis was performed at 5% significance. Missing values are displayed in brackets. All analyses were performed using SPSS Statistics, version 25 (IBM).

Free-text comments were imported into MS Excel 2016 to facilitate the categorisation and indexing of data. Categories were synthesised through inductive content analysis. To ensure reliability, two researchers coded items independently; ambiguities were resolved by discussion.

## Results

### Study participants

In total, 165 out of 897 FDs participated in the survey (response rate: 18.4%). The mean age was 56 ± 7.7 years. Almost all participants were practice owners (96.4%). On average, they had been licensed for the statutory health insurance for 19 ± 9.1 years. A plurality of the FDs worked in group practices (43.7%). For further analysis we included the 142 participants who completed more than 50% of the survey.

### FDs’ experiences with and patient communication regarding self-help groups

Details of respondents ‘answers are presented in Table 1. About two thirds t of our respondents were not aware of any local SHGs. Only 3.2% of the participants reported that patients frequently inquire about SHGs. The majority (60.6%) of FDs reported that they inform their patients about SHGs during consultations (Table 1).

**Table 1:** Characteristics of physicians and their practices (*N* = 165)^ab^

**Physician characteristics**	
Age (years) [11]	
Mean (± SD)	56 (±7.7)
Licensed for the statutory health insurance funds	
Mean (± SD)	19 (±9.1)
Practice owner, (N %) [6]	153 (93)
**Practice characteristics**	
Practice setting, N (%) [6]	
Solo practice	64 (40.3)
Group practice	69 (43.4)
Other	26 (16.4)
Number of patients in practice (quarterly), N (%) [7]	
≤ 1000	29 (18.4)
1001–2000	77 (48.7)
2001–3000	30 (19.0)
> 3000	22 (13.9)
**FDs‘experience with and communication regarding SHG**^**c**^	
Experience with SHG, N (%)	
My patients very often ask about SHGs [10]	5 (3.2)
I know of a local SHG for type 2 diabetics [11]	28 (18.2)
I know of a local SHG for CAD patients [9]	29 (18.6)
Referral to SHGs via, N (%)	
Consultation	91 (56.5)
Flyers, posters or similar	36 (22.4)
Practice assistants	23 (14.3)

*SD* Standard deviation, *FD* Family doctor, *SHG* Self-help group, *CAD* Coronary artery disease

^a^[Missing values]

^b^Based on respondents who “fully agree” and “agree” with the listed statements

Most participants rated the existing lay support for their patients as insufficient (61.9 and 58.5% for T2D and CAD patients, respectively).

### Peer support programs

Overall, most FDs viewedPSPs in a positive light. Almost two-thirds of the physicians surveyed (64.0% T2D, 65.5% CAD) agreed that patients with T2D and CAD would benefit from a peer support group (Table 2).

**Table 2:** FDs’ attitudes toward PSPs (*N* = 142), N (%)^a b^

	Disagree/Strongly Disagree	Neither agree nor disagree	Agree/Strongly Agree
**Attitudes toward SHG**			
Currently available programs of lay support meet the needs of the majority of my T2D patients [8].	83 (61.9)	30 (22.4)	21 (15.6)
My T2D patients would benefit from peer support groups [3].	11 (8.0)	39 (28.1)	89 (64.0)
Currently available programs of lay support meet the needs of the majority of my CAD patients [7].	79 (58.5)	37 (27.4)	19 (14.0)
My CAD patients would benefit from peer support groups [3].	14 (10.1)	34 (24.5)	91 (65.5)
**Attitudes toward PSP**			
I would like to see the materials used in the peer support program [11].	13 (9.9)	13 (9.9)	105 (80.2)
I think that my practice would, in the long run, benefit from a peer support program [9].	43 (32.4)	40 (30.1)	50 (37.6)
I don‘t think a peer support program has significant value added to traditional self-help groups [11].	86 (65.7)	24 (18.3)	21 (16.1)
I consider a peer support program to be a valuable extension to existing care [9].	4 (3.1)	23 (17.3)	106 (79.7)
I would advise my DMP patients to participate in a peer support program [9].	3 (2.3)	11 (8.3)	119 (89.5)
**Target group for PSP**^**c**^			
I would encourage all my T2D patients to participate in the peer support program [10].	–	–	26 (19.7)
I would only approach specific T2D patients [10].	–	–	92 (69.7)
I would encourage all my CAD patients to participate in the peer support program [12].	–	–	15 (11.5)
I would only approach specific CAD patients [12].	–	–	97 (74.6)

*FD* Family doctor, *PSP* Peer support program, *SHG* Self-help group, *T2D* Type 2 diabetes, *CAD* coronary artery disease, *DMP* Disease Management Program

^a^[Missing values]

^b^Based on the respondents who answered at least 50%of the questionnaire

^c^Based on “Yes/No” questions, 2 out of 4 options selectable

Respondents had mixed views regarding the long-term effects of PSP on the their practice workload. About one-third expected a long-term benefit to the practice (37.6%) (Table 2).

### FDs‘attitudes toward the demand and target groups for a PSP

About 89.5% would recommend a program to their patients, both for CAD and T2D (Table 2). However, various pre-requisites were considered important before recommending a PSP. For the domain ‘organisation’, the most important aspects were ‘sufficient flexibility in scheduling‘(69.7%) and ‘participation with partner allowed‘(69.0%). Regarding practice expenses, participants rated ‘financial compensation for accompanying FD care‘(67.6%) and ‘appealing information brochures‘(65.5%) as most important. In the domain ‘participants‘, FDs reported that ‘free access‘(65.5%) was most important. In the domain ‘feedback for practices‘, ‘detailed information on the educational content of the program‘(62.0%) was important to the majority of FDs (see Appendix Supplementary Table S1). Regarding multivariable logistic modelling, the results indicated only age as an independent predictor of FDs’ recommendation to PSP. With advancing age, FDs were less likely to recommend PSPs to their patients (OR: 0.86; 95% CI: 0.77–0.96; *p* = 0.007).

### Composition of Peer Support Groups (PSGs)

Concerning the composition of PSGs, FDs rated medical aspects as the most relevant category (M 1.7 on a scale of 1 to 3), followed by organisational (2.0) and social aspects (2.3) (see Appendix Supplementary Table S2). Regarding medical aspects respondents named ‘exercise capacity‘(2.6 on a scale of 1 to 6) and ‘health literacy‘(2.9) as most important, while the BMI (4.4) was regarded as the least relevant medical aspect to influence group composition. Other aspects included ‘physical mobility‘(3.0),‘comorbidity‘(3.5) and ‘mental resilience‘(4.0) (in decreasing order of importance).

In the category ‘organisational aspects‘, respondents were asked to rank eight predefined aspects. FDs felt that ‘proximity‘(1.4 on a scale of 1 to 8) was the most important, followed by ‘good accessibility by public transportation‘(2.8), ‘convenient meeting times‘(3.4), ‘taking along relatives without illnesses‘(4.0), ‘special target groups‘(e.g., women only) (4.5), ‘possibility of switching groups‘(5.3) and ‘provision of childcare‘(6.2).

In the category ‘social aspects‘, ‘language‘(2.5) and ‘age‘(2.8) were rated as the two most important factors influencing the composition of PSGs. The other aspects, in decreasing order of importance, were ‘education‘(3.1), ‘cultural background‘(3.7), ‘professional status‘(4.6), ‘marital status‘(5.0) and ‘gender‘(5.7) (see Appendix Supplementary Table S3).

### Target group for participation in a PSP

The majority of FDs prefer to identify specific patients for participation in such a program (71.2% % T2D, 76.3% % CAD) rather than issue a general recommendation (Table 2). Participants felt that the following patients would benefit most (in decreasing order, see Table 3):

- Patients with insufficient self-structuring (82.7%),
- Patients with a lack of motivation (76.3%),
- Patients with a lack of social contacts (67.6%),
- Singles (64.7%),
- Patients with poor clinical outcomes (62.6%).

**Table 3:** FDs’ assessment: Patients that would benefit particularly from a PSP (*N* = 142), N (%)^a b^

Patients…	Total
with insufficient self-structuring [3]	115 (82.7)
with lack of motivation [3]	106 (76.3)
with lack of social contacts [3]	94 (67.6)
who are single [3]	90 (64.7)
with poor clinical outcomes [3]	87 (62.6)

^a^[Missing values]

^b^Based on respondents who answered at least 50%of the questionnaire

‘Newly diagnosed‘ and ‘motivated patients’ were additionally mentioned in the free-text field.

### Identifying potential peer support group leaders (PSG-L)

FDs were willing to recruit possible PSG-L (patient volunteers with T2D and/or CAD who receive 24 hours of training) from their patient populations. On average, FDs could identify 4 (± 3.2) potential group leaders, and 80% of FDs could think of at least two group leader candidates. Most FDs were confident that they could easily identify suitable candidates using three characteristics: PSG-L should have a positive attitude (87.2%) a successful ‘patient career‘, i.e., improved their self-management from ‘poor‘to ‘good‘, (82.6%), and strong social skills (78.1%) (Table 4 and Supplementary Table S4).

**Table 4:** FD’s perspective of how well qualities of potential group leaders can be assessed (*N* = 142), N (%)^a b^

A suitable group leader…	Very difficult/rather difficult to asses	Rather easy/easy to asses
has enough available time for group management [10]	• 57 (43.1)	75 (56.8)
has an affinity for technology, e.g., when using an online platform [9]	• 48 (36.1)	116 (87.2)
is highly motivated for group management [9]	• 44 (33.1)	89 (66.9)
has good social skills [14]	• 28 (21.9)	100 (78.1)
has had a successful “patient career” (from “poorly adherent” to “well managed”) [10]	• 23 (17.4)	109 (82.6)
has a positive attitude [9]	• 17 (12.8)	116 (87.2)

^a^[Missing values]

^b^Based on respondents who answered at least 50%of the questionnaire

### Facilitators of and barriers to implementation

FDs reported the following main facilitators for implementing a PSP: ‘motivation through peers‘(92.5%), ‘joint exercise activities‘(79.1%), ‘physical activity‘(73.9%), ‘socialising‘ (70.1%) and ‘telephone support‘ (38.8%). On the other hand, ‘loss of motivation over time‘ (73.1%), ‘participants’ unwillingness to participate’ (70.9%), ‘lack of group cohesion’ (44.0%), and ‘overburdening of group leaders’ (44.0%) were the most frequent barriers to PSP (Table 5).

**Table 5:** FDs’ answers: Barriers to and facilitators for PSP implementation (*N* = 142), N (%)^a b^

Facilitators	Total
**Patient level**	
Mutual motivation through peers	124 (92.5)
Joint exercise activities	106 (79.1)
Inclusion of physical activity	99 (73.9)
Initiation of social contacts	94 (70.1)
**Group level**	
Phone support for patients with increased support needs	52 (38.8)
Integration of PSP in DMP	45 (33.6)
Compensation of physician expenses via DMP	43 (32.1)
Support by experts	43 (32.1)
Teaching of theoretical content	42 (31.3)
Personalised feedback reports	42 (31.3)
Compensation of the time and effort of assistants via DMP	38 (28.4)
Application of evidence-based knowledge	32 (23.9)
Online service	30 (22.4)
Wide range of activities	27 (20.1)
**Barriers**	
**Patient level**	
Patients loose interest in the program	98 (73.1)
Patients cannot be motivated to participate	95 (70.9)
**Group level**	
Lack of group cohesion	59 (44.0)
Overburdening of group leaders	59 (44.0)
Drop-out of the group leaders	55 (41.0)
Travel to the group meeting is too burdensome	46 (34.3)
**Practice level**	
Workload too high for the practice team	34 (25.4)
Not enough feedback for the family doctors	32 (23.9)
Workload for physicians too high	31 (23.1)
Possible negative influence on physicians’ treatment	9 (6.7)

*PSP* Peer support program*, DMP* Disease Management Program

^a^[Missing values]

^b^Based on respondents who answered at least 50%of the questionnaire

### Further information requested on PSPs

Regarding additional information, FDs requested more information on the content of the program (20.0%), e.g., learning content, information materials, or the specific goals set. In addition, they were interested in experience reports or best practice models (14.4%), as well as organisational information such as the exact course of a program (12.2%) (see Appendix Supplementary Table S5).

## Discussion

This study assessed German FDs’ attitudes toward PSPs and their perceptions of potential barriers and facilitators regarding implementation. Most respondents viewed PSPs positively. This finding confirms the results of previous studies in other populations, countries, and cultures. According to Zhong et al. [18], primary care center personnel and their district health bureau leaders had a positive attitude after implementing a PSP for 6 months: peer support served as a bridge between medical staff and their patients. Likewise, in a qualitative study from the UK [23], FDs and other health care personnel expected that such a program would reduce the risk of developing T2D and improve wellbeing.

FDs preferred to offer PSPs to selected patient groups only rather than to all patients. It is not clear whether other studies on PSPs restricted participation in such a way. In a systematic review [24], which included 23 studies on various PSPs, the overall participation rate was 83.4% % (54.8 - 96.7%). However, even with overall participation rates of 90% or more, attendance at group meetings was much lower (e.g. participation in intervention vs. attendance in group meetings was 92.6 and 61.4%, respectively [25], Smith et al. [26] reported a similar drop-off rate. Restricting PSPs to patients who struggle with lifestyle changes may negatively impact the implementation and effectiveness of PSPs, since high motivation is a key success factor for PSPs [17].

FDs’ attitudes toward and practices concerning traditional SHGs as well as expected benefits from PSPs highlight important barriers to and facilitators for the adoption and maintenance of PSPs. Ensuring optimal conditions for the implementation of PSP requires careful attention to context factors [27] particularly organisational and cultural fit [12] . FDs saw several facilitators at the patient and group level. About a quarter expected barriers at the practice level, and overall, expectations of positive long-term effects on the practice were relatively low. This might reflect a critical attitude toward the impact of PSPs on patient behaviour or a generally pessimistic view of innovations in health care delivery. Our respondents mostly perceived existing self-help services as not being suitable for most T2D and CAD patients, and were more open to PSPs. Several previous studies have reported positive attitudes among primary care clinicians toward peer support [28], but they did not ask about actual referral behaviour. Steginga et al. [29] reported low referral rates among clinicians regarding PSGs for cancer patients and hypothesised that this was due to scepticism about the information provided in groups. Our results show that FDs are indeed very sensitive when it comes to the knowledge transfer in such groups. Few FDs reported that patients frequently ask for information about self-help services. This may be indicative of a lack of interest among patients or, more likely, of a failure to systematically include peer support as a topic [30]. It is therefore not clear whether FDs would actually refer patients to PSPs. Somewhat unexpectedly, only few respondents were concerned about the negative influence of PSP on physician treatment. In addition to barriers on the individual level, organisational barriers are an important moderator of implementation outcomes [31]. Organisational barriers have been shown to be a major impediment to other referral-dependent interventions, such as referral of dialysis patients to PSGs [16]. Lorthios-Guilledroit et al. conceptualized such facilitators of program implementation as a “supportive implementation climate”, which occurs if the program is perceived as relevant and does not run against other priorities [32]. Overall, our findings suggest that the relevance of the program was successfully established, but that more attention needs to be given to clear information about the program’s benefits to the clinician and their practice, as well as how to lower organisational barriers to referral.

### Strengths and limitations

Our study has several strengths. To the best of our knowledge, it is the first of its kind in Germany. We surveyed a large sample, while at the same time informing FDs about the concept of PSPs. Also, we were able to gain a nuanced understanding of barriers and opportunities by using different types of questions, including open-ended questions. However, the study should be interpreted in light of its limitations. Ours was an exploratory survey. Even though we included free-text items, a qualitative study may have uncovered additional themes. The sample consisted of physicians in teaching practices who may be more open to innovations than their peers. The survey was conducted during the COVID-19 pandemic, likely reducing our response rate. FDs were asked to evaluate a concept with which they were not familiar; therefore, answers about likely referral behaviour should be interpreted with caution. In addition, the program was introduced as part of the existing DMPs, which limited participation to DMP patients only. This may result in a positive selection bias, as hard-to-reach patients may not be enrolled in DMPs. However, currently up to 98% %are enrolled in the T2D DMP and up to 72% %in the CAD DMP [33], meaning this bias would likely be small.

## Conclusions and perspectives

Overall, FDs appear to be open to supporting PSPs for their patients with T2D and CAD. However, in order for such a program to be sustainable, careful attention to facilitating and hindering factors is necessary, especially in regards to benefits of the program to the providers. We hope that our results will help tailor communication about PSPs to FDs.

## Supporting information

### English translation

See Appendix Table 6.

## Supplementary Information

**Additional file 1: Table S1.** Important aspects before recommending a PSP; “I am most likely to recommend the programme if…”. **Table S2.** What should be considered as most important in the composition of PSGs?. **Table S3.** PSG composition regarding organisational, medical and social factors. **Table S4.** Key characteristics of PSG-L. **Table S5.** Additional information FDs would like to have about the PSPs: Freetext answers (*N* = 90). **Supplementary Table S6.** Survey.

## Data Availability

The datasets generated during and analysed during the current study are not publicly available due to data privacy restrictions but are available from the corresponding author on reasonable request.

## Abbreviations

BP: Blood pressure
CAD: Coronary artery disease
DMP: Disease Management Program
e.g.: For example
et al.: Et aliae
FD: Family doctor
HbA1c: Glycated haemoglobin, haemoglobin A1c
i.e.: Id est
M: Mean
PSG: Peer support group
PSG-L: Peer support group leader
PSP: Peer support program
P-SUP: Personalised Self-Management Support Program
SD: Standard deviation
SHG: Self-help group
T2D: Type 2 diabetes
UK: United Kingdom
vs.: Versus
