# Supplementary material for: Family doctors’ attitudes toward peer support programs for type 2 diabetes and/or coronary artery disease: an exploratory survey among German practitioners
Source: BMC Prim Care. 2022 Aug 31;23:220. doi: 10.1186/s12875-022-01827-3 (PMC9427433; doi:10.1186/s12875-022-01827-3)
Supplement: Supplementary file 1 — Additional file 1: Table S1. Important aspects before recommending a PSP; “I am most likely to recommend the programme if…”. Table S2. What should be considered as most important in the composition of PSGs?. Table S3. PSG composition regarding organisational, medical and social factors. Table S4. Key characteristics of PSG-L. Table S5. Additional information FDs would like to have about the PSPs: Freetext answers (N = 90). Supplementary Table S6. Survey. [file 12875_2022_1827_MOESM1_ESM.docx]

**Appendix**

**Table S1** Important aspects before recommending a PSP; “I am most likely to recommend the programme if…”

| **Practice feedback:** | **N** | **%** |
| --- | --- | --- |
| …I receive information about the educational content of the programme. | 88 | 62.0 |
| ...I am notified about the participation of my patients. | 70 | 49.3 |
| ...I receive feedback on the lifestyle of participating patients in my practice. | 70 | 49.3 |
| **Participants:** | **N** | **%** |
| …the programme is free of charge. | 93 | 65.5 |
| …patients are invited by their health insurance. | 67 | 47.2 |
| …patients receive incentives for participation (e.g. bonus programmes). | 59 | 41.5 |
| **Organisational details:** | **N** | **%** |
| …there are enough options for peer group meetings. | 99 | 69.7 |
| …participation as a couple is allowed. | 98 | 69.0 |
| …there is sponsorship by health insurance companies / non-profit organisations. | 51 | 35.9 |
| **Support for the practice:** | **N** | **%** |
| ...my practice is reimbursed for providing ongoing care. | 96 | 67.6 |
| …there are appealing informational brochures for patients. | 93 | 65.5 |
| …my practice is reimbursed for informing patients about the programme | 33 | 23.2 |

**Table S2** What should be considered as most important in the composition of PSGs?

|  | **Ø ranking*** | **1st** | | **2nd** | | **3rd** | |
| --- | --- | --- | --- | --- | --- | --- | --- |
|  |  | **n / %** | | | | | |
| Medical aspects [10] | 1.7 | 64 | 48.5 | 50 | 37.9 | 18 | 13.6 |
| Organisational factors [11] | 2.0 | 48 | 36.6 | 37 | 28.2 | 46 | 35.1 |
| Social background [12] | 2.3 | 21 | 16.2 | 45 | 34.6 | 64 | 49.2 |
| *mean of all ratings  [Missing values] | | | | | | | |

| **Organisational**  **Factors** | *Overall ranking*  2.0 | | | | | | |
| --- | --- | --- | --- | --- | --- | --- | --- |
|  | *Proximity* | *Accessibility* | *Time* | *Relatives* | *Specific* | *Change* | *Childcare* |
| *Missings* | 9 | 16 | 19 | 14 | 23 | 20 | 40 |
| *Ø ranking* | 1.4 | 2.8 | 3.4 | 4 | 4.5 | 5.3 | 6.2 |
| Proximity=local proximity, Accessibility= Good accessibility by bus and train, Time= time preferences, Relatives= participation with healthy relatives, Specific= specific PSGs (e.g. women), Change= option to change groups, Childcare= childcare offered | | | | | | | |

**Table S3** PSG composition regarding organisational, medical and social factors

| **Medical**  **Aspects** | *Overall ranking*  1.7 | | | | | |
| --- | --- | --- | --- | --- | --- | --- |
|  | *Physical* | *Literacy* | *Mobility* | *Comorbidity* | *Mental* | *BMI* |
| *Missings* | 14 | 15 | 16 | 16 | 26 | 24 |
| *Ø ranking* | 2.6 | 2.9 | 3.0 | 3.5 | 4.0 | 4.4 |
| Physical= physical resilience, Literacy= health literacy, Mobility= physical mobility, Mental= mental resilience, BMI= body mass index | | | | | | |

| **Social**  **background** | *Overall ranking*  2.3 | | | | | | |
| --- | --- | --- | --- | --- | --- | --- | --- |
|  | *Language* | *Age* | *Education* | *Culture* | *Occupation* | *Marriage* | *Gender* |
| *missings* | 14 | 15 | 16 | 16 | 26 | 24 | 35 |
| *Ø Ranking* | 2.5 | 2.8 | 3.1 | 3.7 | 4.6 | 5.0 | 5.7 |
| Language= language skills, Age= age, Culture= cultural background, Occupation= occupational status , Marriage= marital status | | | | | | | |

| **Please indicate how well you feel you can assess the following characteristics of potential PSG-L…** | | | | |
| --- | --- | --- | --- | --- |
|  | *(very) poorly* | | *(very) well* | |
|  | *N/ %* | | | |
| …positive attitude. [9] | 17 | 12.8 | 116 | 87.2 |
| ...successful ‘patient career’, i.e., improved self-management from ‘poor’ to ‘good’. [10] | 23 | 17.4 | 109 | 82.6 |
| …strong social skills. [14] | 28 | 21.9 | 100 | 78.1 |
| ...highly motivated. [9] | 44 | 33.1 | 89 | 66.9 |
| ...is technophile, e.g. when using an online platform. [9] | 48 | 36.1 | 85 | 63.9 |
| ...has enough time. [10] | 57 | 43.1 | 75 | 56.8 |
| [Missing values] | | | | |

**Table S4** Key characteristics of PSG-L

**Table S5**  Additional information FDs would like to have about the PSPs: Freetext answers (N=90)

| **Category*** | **N** | **%** |
| --- | --- | --- |
| ‘On the content of PSPs‘ | 18 | 20.0 |
| ‘On Best-practice models‘ | 13 | 14.4 |
| ‘On organisation of PSPs‘ / ‘No further information‘ each | 11 | 12.2 |
| ‘On details of PSPs‘ / ‘Not applicable‘ each | 7 | 7.8 |
| ‘Only general information‘ | 6 | 6.7 |
| ‘On implementation‘ / ‘on the information materials distributed in PSPs‘ / ‘on PSGL or contact person‘ | 3 | 3.3 |
| ‘On group meetings‘ / ‘feedback from patients‘ / ‘on funding of PSPs‘ | 2 | 2.2 |
| ‘On participation rates‘ | 1 | 1.1 |

*****categorization through inductive content analysis

**Supplementary Table S6: Survey**

| 1. **Personal details:** | | | |
| --- | --- | --- | --- |
| My age | \|_\|_\|years | | |
| I have been a licensed physician since | \|_\|_\|_\|_\| | | |
| My practice is  (multiple answers possible) | □ Solo practice  □ Single group practice (integrated)  □ Single co-Located group practice  □ Single practice with employed physicians  □ Larger system  □ Medical Service Center  Others: _________________________ | | |
| Number of physicians in the practice | \|_\|_\| physicians | | |
| No. of patients per quarter | □ <500 | □ 1501-2000 | □ >3000 |
|  | □ 501-1000 | □ 2001-2500 | □ not specified |
|  | □ 1001-1500 | □ 2501-3000 |  |
| I am… | □ the practice owner  □ an employed physician | | |

| 1. **Your experiences with self-help groups:** | | | | | |  |
| --- | --- | --- | --- | --- | --- | --- |
| Please indicate to what extent you agree with the following statements. | | | | | |  |
|  | **Strongly Disagree** |  |  |  | **Strongly Agree** | |
| I am very often asked by my patients about self-help groups. | □ | □ | □ | □ | □ | |
| I know of a self-help group for **type 2 diabetics** in the vicinity of my practice. | □ | □ | □ | □ | □ | |
| I know of a self-help group for **CHD patients** in the vicinity of my practice. | □ | □ | □ | □ | □ | |
| In my practice I use the following ways to inform patients about self-help groups….  (multiple answers possible) | □ with flyers, posters or similar  □ in the physician consultation  □ with information events  □ by medical assistants*  □ Other:_______________________ | | | | |  |
| *Medical assistant= specialized physicians’ assistant | | | | | |  |

| 1. **Your opinion on the needs of DMP* patients with CHD and/or diabetes type 2 for peer support programmes** | | | | | |  |
| --- | --- | --- | --- | --- | --- | --- |
| 3.1 Please indicate to what extent you agree with the following statements. | | | | | |  |
| Currently available programmes of lay support meet the needs of the majority of my patients... | **Strongly Disagree** |  |  |  | **Strongly Agree** | |
| **…with diabetes typ 2**. | □ | □ | □ | □ | □ | |
| …**with CHD**. | □ | □ | □ | □ | □ | |
| My patients… |  |  |  |  |  | |
| **…** **with diabetes typ 2** would benefit from peer support groups. | □ | □ | □ | □ | □ | |
| … **with CHD** would benefit from peer support groups. | □ | □ | □ | □ | □ | |
| 3.2. Please select one or two terms in each category that you consider important to recommend such a programme to your DMP* patients. | | | | | |  |
| **Practice feedback:**  □ I receive information about the educational content of the programme.  □ I am notified about the participation of my patients.  □ I receive feedback on the lifestyle of participating patients in my practice. | | | | | |  |
| **Participants:**  □ Patients are invited by their health insurance provider.  □ The programme is free of charge.  □ Patients receive incentives for participation (e.g. bonus programmes). | | | | | |  |
| **Organisational details:**  □ Sufficient options for peer group meetings.  □ Participation as a couple is allowed.  □ Sponsorship by health insurance companies / non-profit organisations. | | | | | |  |
| **Effort for the practice:**  □ My practice is reimbursed for informing patients about the programme.  □ My practice is reimbursed for providing ongoing care.  □ Appealing information brochures for patients. | | | | | |  |
| **What else do you consider important?**  □ Other:_______________________ (free text) | | | | | |  |
| *DMP= Disease Management Program | | | | | |  |

| 1. Your view on the composition of peer support groups: |
| --- |
| *Peer support groups* should possibly consist of a fixed group of participants. In addition to the exchange of experiences and psycho-social support, joint exercise activities will also be carried out.  What should be taken into account to put together matching peer support groups?  Please give a ranking in each category. To do so, drag the fields down into the list or simply double-click on the particular field. |
| Social background:  □ Occupational status  □ Graduation  □ Language  □ Cultural background  □ Age  □ Family status  □ Sex  Other: ___________________ |
| Medical aspects:  □ Body Mass Index  □ Mental resilience  □ Physical mobility  □ Health literacy  □ Comorbidity  □ Exercise capacity  Other: ___________________ |
| Organisational details:  □ Proximity of the programme  □ Groups for specific participants (e.g. women)  □ Time preferences  □ Option to include healthy family members  □ Childcare required  □ Opportunity to change the group  □ Easy to reach by bus and train  Other: ___________________ |
| Now put the categories in a ranking order. Please start again with the most important one:  □ Social background  □ Medical aspects  □ Organizational details |

| **5. Which patient groups would you refer for participation in a peer support programme?** |
| --- |
| □ Everyone with type 2 diabetes  □ Everyone with CHD  □ Particular diabetes patients  □ Particular CHD patients |
| **5.1 In your opinion, which patients would benefit most from a peer support programme?** |
| Patients....  □ after hospitalisation  □ with depression  □ who are single  □ with poor clinical outcomes  □ with language difficulties  □ with lack of social contacts  □ with lack of motivation  □ with insufficient self-structuring  □ who have little interest in health topics  □ Other:______________________ |

| **6. Your views on lay support through support groups:** | | | | | |
| --- | --- | --- | --- | --- | --- |
| ***Peer support group leaders*** are diabetes and/or CHD patients who receive a tailored training. They structure the group meetings using training materials. They act as role models and contact persons for the participants. | | | | | |
| **6.1. Please indicate how well you can assess possible peer support group leaders from your practice with regard to the following characteristics.** | | | | |  |
| The prospective PSG-leader… | □ very difficult to assess | □ rather difficult to assess | □ rather easy to assess | □ easy to assess |  |
| …has available time for group management. | □ | □ | □ | □ |  |
| …has a high motivation for group management. | □ | □ | □ | □ |  |
| …has an affinity for technology, e.g. when using an online platform. | □ | □ | □ | □ |  |
| …has a positive attitude. | □ | □ | □ | □ |  |
| …has social skills. | □ | □ | □ | □ |  |
| …had a successful "patient career" (experience from "poor" to "well" doing patient). | □ | □ | □ | □ |  |
| Other:___________________________(free text) | □ | □ | □ | □ |  |
| **6.2 General practitioners can help recruit dedicated peer support group leaders.**  Please give us your assessment: | | | | |  |
| How many patients with CHD and/or diabetes can you think of who could serve as group leaders? | | \|_\|_\| | | |  |

| **7. Please give us your current opinion on the peer support programme:** | | | | | |
| --- | --- | --- | --- | --- | --- |
|  | **Strongly Disagree** |  |  |  | **Strongly Agree** |
| I would like to see the materials used in the peer support programme. | □ | □ | □ | □ | □ |
| I think that my practice will be benefit in the long run through a peer support programme. | □ | □ | □ | □ | □ |
| I don't think the added value of a peer support programme is significant, compared to traditional self-help groups. | □ | □ | □ | □ | □ |
| I consider a peer support programme to be a useful extension of existing care. | □ | □ | □ | □ | □ |
| I would recommend my DMP* patients to participate in a peer support programme. | □ | □ | □ | □ | □ |
| *DMP= Disease Management Program | | | | | |

| **8. Potential supporting and hindering factors:** | |
| --- | --- |
| 8.1 I consider the following aspects of peer support programs for diabetes and CHD patients to be particularly beneficial: | □ Initiating social contacts.  □ Joint exercise activities.  □ Motivation through peers.  □ Wide range of activities.  □ Teaching of theoretical contents.  □ Inclusion of physical activity.  □ Application of evidence-based knowledge.  □ Support by experts.  □ Personalized feedback reports.  □ Online Service.  □ Telephone support for patients with increased support needs.  □ Compensation of my expenses via DMP*.  □ Compensation of the time and effort of my medical assistants** via DMP*.  □ Integration with DMP*.  □ Others: __________________________ |
| 8.2 The main obstacles to the implementation of a peer support programme are: | □ Patient cannot be convinced of participation.  □ Patient loses interest in the programme.  □ Lack of group cohesion.  □ Drop out of the group leaders.  □ Overloading of the group leaders.  □ Rides to the group meetings.  □ Not enough feedback to the general practitioners.  □ Possible negative influence on physician treatment.  □ Too much workload for physicians.  □ Too much workload for the practice team.  □ Others:________________________________ |
| 8.3 Which requirements would have to be met for you to attend a special seminar as an expert and give a presentation there? | [open answer] |
| 8.4 Under which requirements would you make rooms in your practice available for special seminars? | [open answer] |
| 8.5 What other information would you like to receive about the Peer Support Programme? | [open answer] |
| *DMP= Disease Management Program  **Medical assistant= specialized physicians’ assistant | |
